# Practical Medicine

**Published:** 1857-09

**Authors:** 


### PRACTICAL MEDICINE.

Notes on the Use of Glycerine in Consumption. By *Richard Payne Cotton, M. D.*—There is much difference of opinion as to the influence of glycerine upon cases of Consumption. Not a few medical practitioners consider it scarcely, if at all, inferior to cod-liver oil, whilst there are not wanting those in whose hands it has entirely failed.

With a view of testing its effects, I administered it—in doses varying from one to two, and occasionally three drachms, twice a day—to twenty-three of the in-patients of the Consumption Hospital; notes being carefully kept by the Resident Clinical Assistants, Dr. Stone and Dr. Sibbald. As the cases were not selected, and all of them were under the same dietetic and general hygienic conditions, the result, as exhibited in the following table, may, I think, be regarded as a fair illustration of its influence. (*See opposite page.*)

It will be observed that only in five cases was there any improvement; in all of which the weight was slightly increased. In two of these, however, a much greater advantage was subsequently gained under the use of cod-liver oil; the weight of one patient having increased as much as two pounds per week.

In seventeen cases either there was no appreciable improvement, or the patients became worse; and one, in an advanced stage of the disease, ended fatally. In nine of these cases more or less improvement occurred from the after-use of the oleum aselli; in four instances, indeed (Nos. 4, 12, 19, 22), the gain in weight was very distinctly marked.

In five cases the glycerine either caused sickness, or otherwise disagreed with the stomach.

To any objection which may be raised that the glycerine was not given for a sufficiently long period, I would merely observe, that even in the cases where some improvement was noticeable, it appeared to me so probable that far greater good would accrue from the cod-liver oil, that I regarded a further trial of the other as unjustifiable; and, that such an anticipation was, in some instances at least, not ill-founded, the table sufficiently demonstrates.

| No. | Sex. | Age. | Stage of Disease. | Complications. | Time under treatment. | General effect. | Effect upon weight. | Average gain in weight per week. | REMARKS. |
|---|---|---|---|---|---|---|---|---|---|
| 1 | M. | 41 | 2 | — | 4 weeks. | None. | Gain. | ½ lb. | Had previously been improving under cod-liver oil. |
| 2 | M. | 40 | 2 | Emphysema. | 6 weeks. | None. | Loss. | — | Caused sickness. Patient improved and gained weight afterwards, under cod-liver oil. |
| 3 | M. | 24 | 1 | — | 7 weeks. | None. | Loss. | — | —— |
| 4 | M. | 29 | 1 | — | 3 weeks. | None. | Loss. | — | Afterwards gained 1½ lb. per week under cod-liver oil. |
| 5 | M. | 35 | 3 | Bronchitis. | 10 weeks. | Improvement. | Gain. | 1 lb. | Took iodide of iron also. |
| 6 | M. | 31 | 3 | — | 2 weeks. | None. | Loss. | — | An unpromising case, cod-liver oil having previously failed. |
| 7 | M. | 30 | 3 | — | 2 weeks. | None. | Loss. | — | Ditto. |
| 8 | M. | 16 | 2 | Hæmoptysis. | 3 weeks. | None. | Gain. | ¼ lb. | Caused sickness. Patient afterwards improved under cod-liver oil. |
| 9 | M. | 18 | 2 | — | 7 weeks. | Improvement. | Gain. | ¾ lb. | —— |
| 10 | M. | 34 | 2 | — | 3 weeks. | None. | None. | — | Took steel with the glycerine. |
| 11 | M. | 28 | 3 | — | 3 weeks. | None. | None. | — | Became dyspeptic. Patient afterwards improved under cod-liver oil. |
| 12 | M. | 19 | 1 | — | 3 weeks. | None. | Loss. | — | Afterwards gained 3 lbs. in four weeks under cod-liver oil. |
| 13 | M. | 13 | 3 | — | 2 weeks. | None. | Loss. | — | An unpromising case, other treatment having previously failed. |
| 14 | M. | 19 | 1 | Bronchitis. | 2 weeks. | Improvement. | Gain. | 1 lb. 4 oz. | Afterwards gained at increased rate under cod-liver oil. |
| 15 | F. | 12 | 3 | — | 4 weeks. | Slight improvement. | Gain. | 1 oz. | Health generally improved. |
| 16 | F. | 46 | 3 | — | 4 weeks. | Became worse. | Loss. | — | An unpromising case, other treatment having previously failed. |
| 17 | F. | 25 | 2 | — | 4 weeks. | None. | Loss. | — | Ditto. |
| 18 | F. | 24 | 3 | — | 4 weeks. | Became worse. | Loss. | — | Ditto. |
| 19 | F. | 29 | 1 | — | 4 weeks. | Slight improvement. | Gain. | 4 oz. | Afterwards gained 2 lbs. per week under cod-liver oil. |
| 20 | F. | 30 | 2 | — | 2 weeks. | Died. | Loss. | — | Caused sickness and loss of appetite. |
| 21 | F. | 38 | 2 | — | 3 weeks. | None. | Loss. | — | Ditto. Patient afterwards improved slightly under cod-liver oil. |
| 22 | F. | 24 | 2 | Diarrhœa. | 2 weeks. | None. | Gain. | ½ lb. | Afterwards gained more than 2 lbs. per week under cod-liver oil. |
| 23 | F. | 47 | 2 | — | 1 week. | Became worse. | Loss. | — | Caused gastric disturbance, thirst, and loss of appetite. |

The following conclusions are, I think, irresistible, viz:

1. That glycerine has generally but little influence upon phthisical cases.

2. That, as a remedial agent in consumption, it will bear no comparison with cod-liver oil.—*Med. Times and Gazette, June* 27, 1857.

2. LACTIC ACID A REMEDY FOR DYSPEPSIA.—A remedy which has for a long time been used by Dr. NELSON, of Birmingham, and subsequently by many French physicians, under the name of Pepsine, for the cure of dyspepsia, and other functional derangements of the stomach, has within a short time been prescribed freely by some physicians in London. It has been very favorably noticed by Drs. Ballard and Sieveking. Dr. O'Connor has also tested its value in those cases in which it has been recommended, but not with the success attributed to its use. He was led subsequently to have recourse to lactic acid, a remedy which he believed likely to be more beneficial in those affections of the stomach in which the so-called pepsine has been administered. Before using the acid internally, Dr. O'Connor, we understand, in order to test its digestive powers as compared with pepsine, placed an equal weight of animal fibre, in equal proportions of pepsine and lactic acid, in separate vessels, in an equal temperature, when he found that the fibre in the lactic acid was reduced to a pulpy state in a very much smaller space of time than that which was put into the pepsine. After this experiment, which he thought sufficiently conclusive of the superiority of the lactic acid as a promoter of digestion, he had recourse to its use as a remedy in those affections of the stomach before alluded to. The great number of patients with affections of the stomach presenting themselves among the out-patients of the Royal Free Hospital, afforded an extensive field to Dr. O'Connor for testing the efficacy of lactic acid in dyspeptic conditions. After a trial in over fifty cases, he considers that the good results following its use fully justify him in recommending it as a valuable agent. It is very necessary to be sure that the lactic acid prescribed should be of chemical purity, and of uniform strength. The dose varies from half a drachm to two drachms or more, in infusion of colomba, or a little cinnamon-water. It should be taken during a meal. The lactic acid found in the shops is not generally pure; that which Dr. O'Connor has found to be most efficient, from its greater purity, is prepared by Mr. Bastick, of Brook Street, Grosvenor Square.—*Med. Times and Gaz.*, April 25, 1857.

